# Invasive group A streptococcal (iGAS) surveillance in Island Health, British Columbia, 2022

**DOI:** 10.14745/ccdr.v49i78a06

**Published:** 2023-08-01

**Authors:** Andrea Nwosu, Andrea Schut, Christie Arlotti Wood, Christine Urquhart, Claudia Bachman, Katelyn Thompson, Julia Evans, Kathleen Mills, Lisa Wenstob, Theresa Restemeyer, Trista Galbraith, Shannon Mason, Stephanie Gabriel, Twyla Gasper, Cheryl Broeren, Francine Lewis, Dee Hoyano, Sandra Allison, Pamela Kibsey, Angela Reid, Maritia Gully, Carl Swanson

**Affiliations:** 1 Canadian Field Epidemiology Training Program, Public Health Agency of Canada, Ottawa, ON; 2 Island Health, Victoria, BC

**Keywords:** iGAS, group A streptococcus, *Streptococcus pyogenes*, *emm*, surveillance, British Columbia, Canada

## Abstract

**Background:**

Invasive group A streptococcal disease (iGAS) is caused by *Streptococcus pyogenes* group A bacteria. In 2022, multiple disease alerts for iGAS in the Island Health region, in the context of increased infections in the paediatric population in Europe and the United States, prompted further investigation into local trends. This surveillance study summarizes epidemiological trends of iGAS in the region covered by Island Health, a regional health authority in British Columbia, in 2022.

**Methods:**

In British Columbia, iGAS is a reportable disease; all confirmed cases are reported to the regional authority and the provincial health authority (BC Centre for Disease Control). Island Health’s iGAS surveillance system is passive and collects information on cases that are identified through laboratory testing. Surveillance data were summarized for 2022 and compared with historical data from 2017–2021.

**Results:**

In 2022, the incidence rate was 11.4 cases per 100,000 population (n=101), the highest observed rate in the last six years. The median age of cases was 53 years, with a range of 0–96 years, and 64% of cases were male. The highest risk of infection was reported in men 40–59 years of age, with an incidence rate of 21.3 cases per 100,000 population. The most common *emm* types were *emm*92 (n=14), *emm*49 (n=13), and *emm*83 (n=12). Overall, 85% (n=86) of cases were hospitalized, 21% (n=21) were admitted to the intensive care unit, and 6% (n=6) died.

**Conclusion:**

This study highlights that the incidence of iGAS in the Island Health region continued to increase throughout the coronavirus disease 2019 (COVID-19) pandemic, reaching its highest annual rate in 2022. In contrast to reports from Europe and the United States, there was no notable increase in infections in the paediatric population. Given the sustained increase in iGAS activity, continued monitoring and description of the epidemiology of these cases on a regular basis is imperative.

## Introduction

Group A streptococcal disease (GAS) is caused by *Streptococcus pyogenes* group A bacteria ((1)). A GAS infection is considered invasive when bacteria is detected at a sterile site within the body ((1)). Invasive group A streptococcus (iGAS) causes severe and in some cases life-threatening illness ((1)). In 2022, multiple disease alerts for iGAS in Island Health, a regional health authority in British Columbia, in the context of reports of increased infections in the pediatric population in Europe and the United States, prompted further investigation into local trends ((2,3)). The following surveillance report summarizes epidemiological trends of iGAS in Island Health, British Columbia in 2022.

## Methods

### Population

**Figure 1:**
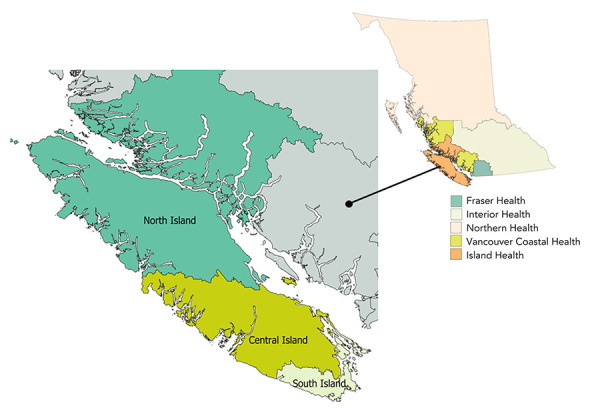
Island Health Region of British Columbia

Island Health is one of five regional health authorities in British Columbia. The Island Health region has a population of about 860,000 people, which includes residents of Vancouver Island, the Islands in the Salish Sea and the Johnstone Straight, and the mainland communities north of Powell River and south of Rivers Inlet (**Figure 1**) ((4)). The region is divided into three health service delivery areas (HSDAs): North, Central and South Island.

## Case definitions

### Confirmed case

Laboratory confirmation of infection with or without clinical evidence of invasive disease: isolation of group A streptococcus (*S. pyogenes*) from a normally sterile site, or demonstration of *S. pyogenes* DNA by an appropriately validated nucleic acid test from a normally sterile site ((5)).

### Probable case

Clinical evidence of invasive disease in the absence of another identified etiology and with non-confirmatory laboratory evidence of infection: isolation of group A streptococcus from a non-sterile site, or positive group A streptococcus antigen detection ((5)).

## Surveillance methods

In British Columbia, iGAS is a reportable disease; all confirmed cases are reported to the regional health authority and then to the BC Centre for Disease Control (BCCDC). Island Health’s iGAS surveillance system is a passive case-based system that relies on the collection of information about cases that are identified through laboratory testing. Laboratory testing of iGAS is conducted locally at Island Health laboratories. Positive bacterial cultures are then sent to the BCCDC Public Health Laboratory for confirmatory testing. Subtyping (*emm* typing) of all isolates is conducted by the Canadian National Microbiology Laboratory (NML). Information on case demographics, clinical progression of illness, and risk factors are collected using a standardized surveillance form.

Island Health case-level data were extracted from BCCDC’s Public Health Reporting Data Warehouse on February 1, 2023, at 12:00 p.m. PST. The case line list included episode date and information on age, sex, risk factors, and outcomes. The episode date is equal to the onset date if available. If the onset date is not available, then the clinical diagnosis date is used, followed by the earliest of specimen collection date, laboratory result date, or report date.

## Data analysis

All analyses were performed using R version 4.1.1 and RStudio version 1.4.1717. Trends in case counts, incidence rates, geographic distribution, demographics, severity, and risk factors were summarized for 2022 and compared with historical data from 2017–2021. Population denominators were used to calculate rates.

## Results

### Trends in case counts and rates

**Figure 2:**
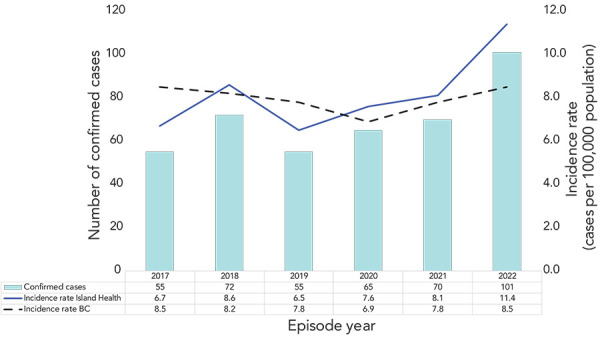
Invasive group A streptococcal disease cases and incidence rates by year, Island Health, 2017–2022 (n=418) Abbreviation: BC, British Columbia Note: The provincial incidence rates provided in this figure are preliminary as of January 27, 2023. They are subject to change after the data reconciliation process has been completed by BC Centre for Disease Control

Incidence rates of iGAS in the Island Health region have been increasing since 2019 (**Figure 2**). From 2017 to 2022, incidence rates ranged from 6.7 cases to 11.4 cases per 100,000 population. In 2022, 101 confirmed cases of iGAS were reported in the Island Health region. The incidence rate was 11.4 cases per 100,000 population, which was above the preliminary annual provincial rate (8.5 cases per 100,000 population) and the highest observed incidence in the last six years.

**Figure 3:**
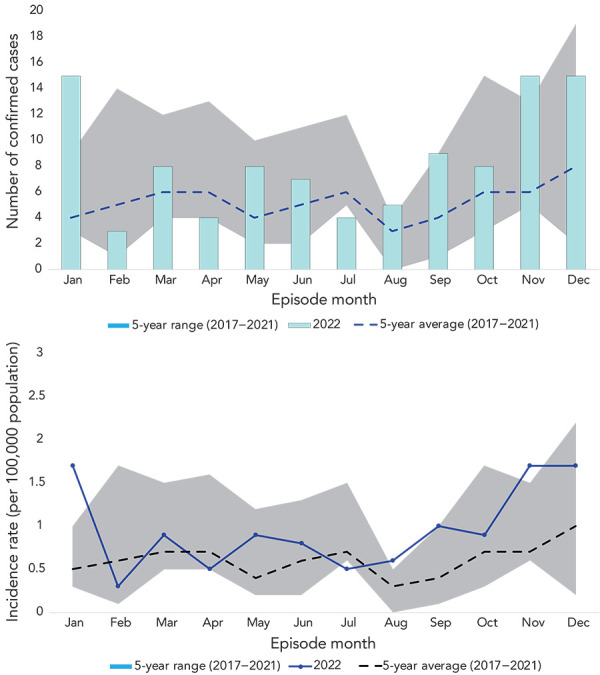
Invasive group A streptococcal disease cases and incidence rates by month, Island Health, 2022 compared to 2017–2021 Note: There is an issue of small numbers when breaking down cases by month. Calculated rates where the numerator is less than 20 are unstable and should be interpreted with caution. Fluctuations in these values may indicate random variation rather than significant change in the rate

The number of reported cases ranged from 3–15 cases per month (incidence range: 0.3 cases to 1.7 cases per 100,000 population) (**Figure 3**). The highest observed cases and monthly incidence rates were in January, November, and December (15 cases, incidence rate: 1.7 cases per 100,000 population). In January and November, the number of cases and incidence rate exceeded the maximum cases and incidence seen in the previous five years. The number of cases in these months were 2.5 times and 1.9 times the maximum number of cases reported in the previous five years.

## Geographic distribution

**Figure 4:**
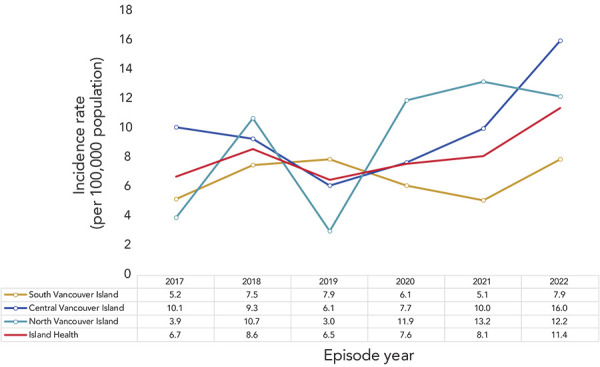
Invasive group A streptococcal disease incidence rates by health service delivery area and year, Island Health, 2017–2022 Note: There is an issue of small numbers when breaking down cases by the health service delivery areas (HSDA), specifically for North Vancouver Island. Calculated rates in this HSDA are based on numerators with fewer than 20 cases. Therefore, these rates are unstable and should be interpreted with caution. Fluctuations in these values may indicate random variation rather than significant change in the rate

The incidence rates in 2022 ranged from 7.9 to 16.0 cases per 100,000 population in the three HSDAs (**Figure 4**). The incidence rates in both North and Central Island exceeded the rates for the entire Island Health Region. Since 2019, the incidence rates in Central Island have been increasing. In North Island, the incidence rates increased from 2019 to 2021 and decreased in 2022. In South Island, the incidence rates decreased from 2019 to 2021 and increased in 2022. In 2022, the highest incidence rate occurred in Central Island at 16.0 cases per 100,000 population. Forty-nine cases were reported from Central Island, which is an increase of 19 cases (63% increase) compared to the number reported in the previous year.

## Demographic distribution

**Figure 5:**
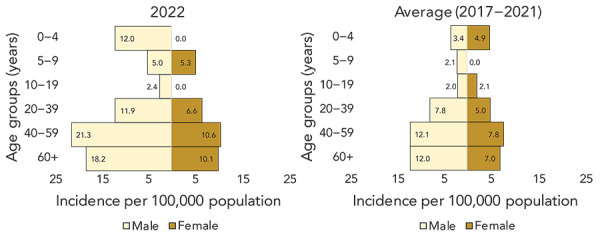
Invasive group A streptococcal disease cases and incidence rates by age and sex, Island Health, 2022 compared to 2017–2021 Note: There is an issue of small numbers when breaking down cases by age group and sex. Calculated rates where the numerator is less than 20 are unstable and should be interpreted with caution. Fluctuations in these values may indicate random variation rather than significant change in the rate

The median age of cases was 53 years, with a range of 0–96 years and 64% of cases were male. The distribution and risk of infection were the highest in men (distribution: 64%, incidence: 15.0 cases per 100,000 population) and individuals 40 years of age and older (distribution: 76%, incidence: 14.7 cases per 100,000 population) (**Table 1**). The highest incidence was reported in men 40–59 years of age (21.3 cases per 100,000 population) (**Figure 5**).

**Table 1:** Invasive group A streptococcal disease cases, distribution, and incidence by age and sex, 2022 compared to 2017–2021

Demographics	2022			Average (2017–2021)		
	Number of cases	Distribution	Incidence rate (per 100,000 population)	Number of cases	Distribution	Incidence rate (per 100,000 population)
**Age group (years)**						
0–4	2	2%	6.1	1	2%	4.1
5–9	2	2%	5.1	0	1%	1.1
10–19	1	1%	1.2	2	3%	2.1
20–39	20	20%	9.3	13	21%	6.4
40–59	35	35%	15.8	22	34%	9.9
60+	41	41%	13.9	25	40%	9.3
**Sex**						
Female	36	36%	8.0	25	40%	5.9
Male	65	64%	15.0	38	60%	9.2

Note: There is an issue of small numbers when breaking down cases by age group and sex. Calculated rates where the numerator is less than 20 (i.e. for age groups younger than 20 years of age) are unstable and should be interpreted with caution. Fluctuations in these values may indicate random variation rather than significant change in the rate

## *Emm* typing

In 2022, there was no single dominant *emm* type. The three most common reported *emm* types were *emm*92 (n=14), *emm*49 (n=13), and *emm*83 (n=12) (**Table 2**).

**Table 2:** Distribution of *Streptococcus pyogenes emm* types by year, Island Health, 2017–2022

*Emm* type	2017	2018	2019	2020	2021	Average (2017–2021)	2022
*emm*92	1	0	0	0	8	2	14^a^
*emm*49	0	0	0	1	15^a^	3	13^a^
*emm*83	0	2	4	4	2	2	12^a^
*emm*74	0	0	1	0	1	0	9
*emm*59	0	0	0	5	6	2	8
*emm*43	0	0	0	0	2	0	6
*emm*76	1	16^a^	2	5	1	5	4
*emm*53	3	3	4	4	1	3	3
*emm*12	0	1	0	1	0	0	3
*emm*11	1	0	1	0	2	1	2
*emm*77	4	0	3	2	4	3	2
*emm*82	6^a^	2	1	1	1	2	2
*emm*1	3	11	6	1	0	4	1
*emm*101	2	2	3	7^a^	3	3	1
*emm*22	1	0	0	0	0	0	1
*emm*41	7^a^	16^a^	10^a^	4	1	8	1
*emm*89	4	2	0	1	1	2	1
*emm*81	0	3	1	3	0	1	1
*emm*114	0	0	0	0	0	0	1
*emm*104	1	0	0	0	0	0	0
*emm*2	3	0	0	0	0	1	0
*emm*28	2	1	1	1	0	1	0
*emm*3	1	0	0	0	0	0	0
*emm*4	1	1	2	0	0	1	0
*emm*73	1	0	0	0	0	0	0
*emm*87	1	0	0	0	0	0	0
*emm*91	1	3	1	0	0	1	0
*emm*118	0	0	1	0	0	0	0
*emm*78	0	0	1	1	0	0	0
*emm*6	0	0	0	5	0	1	0
*emm*68	0	0	0	2	0	0	0
*emm*9	0	0	0	1	0	0	0
*emm*51	0	0	0	0	1	0	0
*emm*75	0	0	0	0	2	0	0
Unknown	11	9	13	16	19	14	16

^a^ Highlighted values indicate the most common *emm* types for each year

## Severity

Twenty-seven percent of cases reported in 2022 were clinically classified as severe (**Table 3**). Severe cases are defined as cases of streptococcal toxic shock syndrome (STSS), soft-tissue necrosis (including necrotizing fasciitis, myositis, or gangrene), meningitis, GAS pneumonia, or death directly attributable to GAS infection ((6)). Overall, 85% of cases were hospitalized, 21% were admitted to the intensive care unit (ICU), and 6% died (**Table 4**). The proportion of cases admitted to the hospital and ICU was below the average number admitted in the previous five years (hospitalizations: average=90%, range=85%–93%; ICU admissions: average=23%, range=15%–32%). The case fatality rate was the same as the average case fatality rate reported in the previous five years (average=6%, range=4%–8%). The deaths reported in 2022 occurred in men and women 52–89 years of age (median age=73 years, 67% female). All cases had multiple risk factors reported (median number of reported risk factors=4, range=2–5). Five different *emm* types were prevalent amongst these fatal cases: 74, 81, 83, 92, and 43.

**Table 3:** Invasive group A streptococcal disease cases and distribution by severity, 2022 compared to 2017–2021

Severity	2022		Average (2017–2022)	
	Cases	Distribution	Cases	Distribution
Severe	27	27%	13	21%
Non-severe	74	73%	38	60%
Unknown	0	0%	12	19%

**Table 4:** Invasive group A streptococcal disease cases and distribution by outcomes, 2022 compared to 2017–2021

Outcomes	2022		Average (2017–2022)	
	Cases	Distribution	Cases	Distribution
Hospitalizations	86	85%	57	90%
ICU admissions	21	21%	15	23%
Deaths	6	6%	4	6%

Abbreviation: ICU, intensive care unit

**Figure 6:**
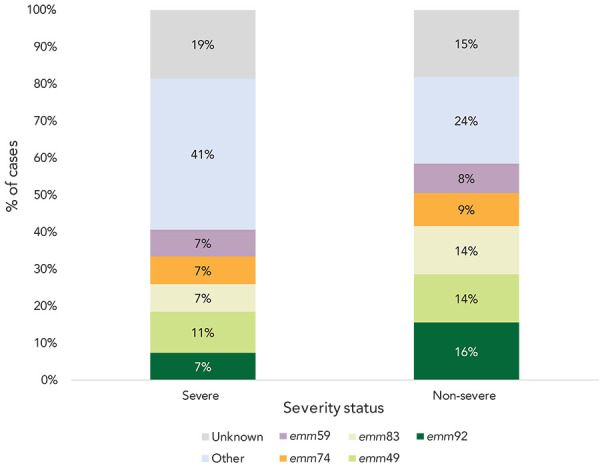
Distribution of streptococcal pyogenes *emm* types by severity, Island Health, 2022

There was no dominant *emm* type reported among severe cases. For both severe and non-severe cases, the most common *emm* types were the same (**Figure 6**).

## Risk factors

The most common reported risk factors among cases were having a skin infection, 47% (n=47) and having a wound, 46% (n=46) (**Table 5**). Compared to the previous five years, skin infections, wounds, alcohol use disorder, unstable housing, chronic cardiac conditions, chronic respiratory conditions, and immunocompromised conditions were reported more frequently in 2022, while injection drug use was reported less frequently. Among severe cases (n=27), the most common reported risk factors were having a wound, 52% (n=14); using substances, 52% (n=14); or having a skin infection, 44% (n=12) (**Table 6**). For non-severe cases (n=74), the most common reported risk factors were having a skin infection, 47% (n=35) or having a wound, 43% (n=32).

**Table 5:** Risk factors reported among cases of invasive group A streptococcal disease cases, Island Health, 2022 compared to 2017–2022

Risk factors	2022		Average (2017–2022)	
	Cases	Distribution	Cases	Distribution
Skin infection	47	47%	24	38%
Wound	46	46%	22	34%
Substance use^a^	40	40%	25	39%
Chronic cardiac condition	31	31%	13	21%
Homeless/underhoused	27	27%	14	23%
Alcohol use disorder	26	26%	14	22%
Chronic respiratory condition	21	21%	9	14%
Diabetes	19	19%	11	17%
Injection drug use	17	17%	14	22%
Immunocompromised	12	12%	5	8%
Substance use, other^b^	2	2%	1	2%
Tobacco use	1	1%	1	1%

^a^ Substance is a composite variable that includes alcohol use disorder, injection drug use, tobacco use, and substance use, other

^b^ Substance use, other is a variable used to capture any other type of substance use than the ones available for selection in the online data reporting system (i.e. alcohol use disorder, tobacco use and injection drug use)

**Table 6:** Risk factors reported among cases of invasive group A streptococcal disease cases, by severity status, Island Health, 2022

Risk factors	Severe	Non-severe
	n=27	n=74
Wound	52%	43%
Substance use^a^	52%	35%
Skin infection	44%	47%
Chronic cardiac condition	37%	28%
Alcohol use disorder	37%	22%
Chronic respiratory condition	30%	18%
Diabetes	26%	16%
Homeless/underhoused	26%	27%
Immunocompromised	22%	8%
Injection drug use	19%	16%
Tobacco use	0%	1%
Substance use, other^b^	0%	3%

^a^ Substance is a composite variable that includes alcohol use disorder, injection drug use, tobacco use, and substance use, other

^b^ Substance use, other is a variable used to capture any other type of substance use than the ones available for selection in the online data reporting system (i.e. alcohol use disorder, tobacco use and injection drug use)

## Discussion

In 2022, 101 confirmed cases of iGAS were reported in the Island Health region, corresponding to an incidence rate of 11.4 cases per 100,000 population; the highest rate reported in the last six years and above the preliminary annual provincial rate (8.5 cases per 100,000 population). Since 2019, the incidence of iGAS has been increasing in the Island Health region. This includes throughout the pandemic period when implemented non-pharmaceutical containment measures were also associated with a decrease in invasive respiratory diseases worldwide ((7)). Provincially, in British Columbia, rates of iGAS have been higher than expected since 2017, with the incidence in the last six years remaining stable ((8)). Globally, an increase in the incidence of iGAS over time has also been observed in many countries, including Canada ((9–12)). Previous analyses have hypothesized that the observed increase is linked to both the increase in genetic diversity of circulating *emm* types and compounding societal risk factors, such as homelessness and substance use ((10,13–17)). Although the factors associated with the increased incidence seen in the Island Health region since 2019, and particularly in 2022, are not completely clear, it is likely that multiple factors have contributed to the observed trends. This includes increased circulation of respiratory viruses, an increase in the diversity in circulating *emm* types, and the impact of the coronavirus disease 2019 (COVID-19) pandemic on community services, specifically an increased demand paired with reduced capacity and availability.

In December 2022, several European countries and the United States reported recent increases in infections of iGAS in children ((2,3)). Similar to the provincial picture in British Columbia, demographic analysis of Island Health cases showed no notable increase in infections among the paediatric population ((8)). The highest risk of infection was observed in men 40 years of age and older. While men 40 years of age and older appear to be at a higher risk for iGAS in 2022, further analysis on iGAS in this demographic group would contribute to understanding whether this is a confounding factor, since other risk factors, such as substance use, are known to be higher in this population ((18–20)).

In 2022, no single dominant *emm* type was identified in the Island Health region. The three most common reported *emm* types were *emm*92 (n=14), *emm*49 (n=13), and *emm*83 (n=12). Prior to 2021, these *emm* types were uncommon in the Island Health region and British Columbia, representing on average 0.4%–4% and 1% of subtyped cases reported from 2016 to 2020, respectively ((8)). Nationally, *emm*1 has been the dominant *emm* type for the last decade ((21)). Since 2014, the prevalence of *emm*1 has been decreasing nationally and was surpassed by *emm*76 in 2019 and *emm*49 in 2020 ((9,22–24)). To date in the available literature, *emm* types 49, 83, and 92 have not been associated with more life-threatening illness. *Emm* types 1 and 3 have been associated with more life-threatening illness, but only represented 1% of cases subtyped in the Island Health region in 2022 ((25–27)). Overall, indicators of severity in the Island Health region were either below the average or within range of the values reported in the previous five years.

## Limitations

When breaking down case numbers by subgroups, cell sizes become small. Calculated rates where the numerator is less than 20 are unstable and should be interpreted with caution. The descriptive analyses where cases are broken down by month, by HSDA (applies to North Island), by age (applies to age categories younger than 20 years of age), and by age and sex are affected by small cell sizes. Fluctuations in these values may indicate random variation rather than significant change in the rate. As well, information on risk factors is predominantly collected through chart reviews. These reviews may not capture the full medical or social history of each case, therefore risk factors among iGAS cases may be under-reported. The regional data presented in this report have undergone data quality assessment by Island Health, but data reconciliation processes for the provincial data are underway for cases reported for 2019 through 2022. The provincial rates shown are based on preliminary numbers, and final numbers and rates for the province may change. Lastly, this report includes data from pandemic response years and an analysis on the impact of the response on the completeness and trends of respiratory surveillance data in the Island Health region has not yet been conducted. It is likely that due to the response, both burden of disease and data completeness decreased, therefore, observed trends during these years might have been higher than reported in this publication. This would affect the interpretation of observed trends in 2022 in comparison to the previous five years. Despite these limitations, this summary contributes descriptive epidemiology that is important for understanding iGAS in the Canadian context.

## Conclusion

Overall, this surveillance study characterizes cases of iGAS in the Island Health region in 2022 and compares these cases to those reported over the last five years. The study highlights that incidence of iGAS in the Island Health region continued to increase throughout the COVID-19 pandemic, reaching its highest annual rate in 2022. In contrast to reports from Europe and the United States, there was no notable increase in infections in the paediatric population. The findings of this report contribute to the epidemiological characterization of iGAS in Canada. Given the continued local, provincial, and national increase in incidence of iGAS, it is imperative that the epidemiology of these cases continues to be monitored and described annually.
